# Pronounced response of papillary craniopharyngioma to treatment with vemurafenib, a BRAF inhibitor

**DOI:** 10.1007/s11102-015-0663-4

**Published:** 2015-06-27

**Authors:** Simon J. B. Aylwin, Istvan Bodi, Ronald Beaney

**Affiliations:** 1 Department of Endocrinology, King’s College Hospital, London, SE5 9RS UK; 2 Department of Clinical Neuropathology, King’s College Hospital, London, UK; 3 Department of Neuro-oncology, Guy’s & St Thomas’ Hospital Cancer Centre, London, UK

The BRAF V600E mutation has recently been identified in a high percentage of papillary craniopharyngiomas [1, 2]. The V600E mutation constitutively activates the MEK-ERK pathway, and the mutation-specific BRAF inhibitor vemurafenib, which targets mutated BRAF, is effective in malignant melanoma [3]. The use of vemurafenib in malignancy now includes hairy cell leukaemia which frequently carries the same mutation, thereby demonstrating that the agent targets the pathway rather than the specific tumor type [4]. Papillary craniopharyngiomas are histologically benign but cause substantial morbidity due to visual loss, pituitary dysfunction, diabetes insipidus and hypothalamic disturbance. We report the first successful use of the BRAF inhibitor vemurafenib in a patient with progressive visual failure due to a recurrent papillary craniopharyngioma, thereby supporting the evidence that the BRAF V600E mutation is pathological in this condition.

**Fig. 1:**
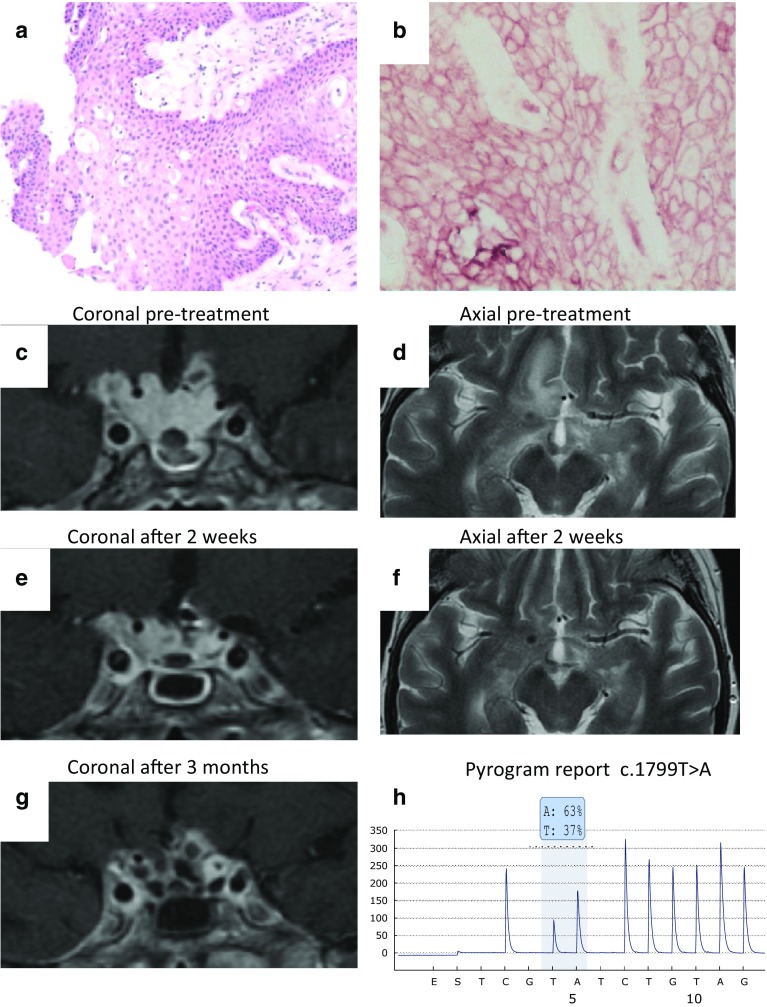
Representative histological sections illustrating (**a**) a papillary craniopharyngioma which is predominantly composed of fibrovascular stroma surrounded by squamous epithelium without formation of keratin pearls (haematoxylin-eosin). (**b**) Immunohistochemistry for β-catenin reveals membranous positivity, characteristic of papillary variant of craniopharyngioma. Pyrosequencing analysis (**h**) indicates the presence of the BRAF mutation c.1799T > A (p.Val600Glu) present in 37 % frequency. Coronal T1 post contrast MRI images through the sella (**c**) pretreatment, (**e**) 2 weeks and (**g**) 3 months after initiation of vemurafenib 960 mg bd demonstrating a largely solid, sellar and suprasellar 30 mm mass with irregular outline invaginating into the right temporal lobe with interval reduction in the volume of residual craniopharyngioma. Axial T2 images pretreatment (**d**) and 2 weeks after initiation (**f**) demonstrate associated reduction in oedema in the left gyrus rectus and right medial temporal lobe

The patient initially presented aged 27 years in 1984 with galactorrhea, amenorrhea, hyperprolactinemia and a pituitary mass. Due to tumor progression while receiving bromocriptine she underwent trans-sphenoidal surgery (TSS) in 1992 with radiological remission but without a conclusive histological diagnosis. She was seen again in 2010 with bitemporal hemianopia and recurrent tumor, and underwent a second TSS followed by fractionated radiotherapy. Histology showed a papillary craniopharyngioma (Fig. 1a, b). There was further tumor progression and despite a third TSS (June 2014) she experienced progressive visual deterioration (RE: no perception of light; LE temporal hemianopia 6/60). The BRAFV600E mutation was confirmed in the tumor (Fig. 1h), and with institutional permission treatment with vemurafenib 960 mg bd was initiated and continued for 3 months.

The pretreatment MRI (Fig. 1c) showed an invasive sellar and suprasellar mass with perifocal edema (Fig. 1d). After 2 weeks on vemurafenib her vision stabilized and MRI showed a marked reduction in the size of the tumor with resolution of the surrounding edema (Fig. 1e, f). Three months after starting treatment there was near-complete resolution of the craniopharyngioma (Fig. 1g). Associated with tumor size reduction, she developed a CSF leak, pneumocephalus and meningitis, necessitating antimicrobial therapy and surgical repair. This is a recognised complication rarely observed when a prolactinoma shrinks with dopamine agonist therapy [5]. Her vision improved and stabilized (RE NPL; LE 6/24) but she suffered frontal lobe volume loss with reduced cognitive status. Treatment was interrupted after 3 months but the tumor recurred within 6 weeks (not shown) and she restarted therapy with vemurafenib. Her tumor exhibited a further response and growth was stabilised until 7 months after treatment initiation when progressive regrowth was observed.

The discovery of the activating BRAF mutation in melanoma and the development of molecular targeted therapy represented a major advance in the management of melanoma. Our case now extends the potential use of vemurafenib to a different indication. Craniopharyngioma is a benign non-metastatic tumor with a low proliferation rate that grows slowly but inexorably and invades surrounding tissues. Treatment mainstays include either trans-sphenoidal or trans-cranial surgery, with external beam radiotherapy. This tumor frequently arises during childhood and the use of radiotherapy has to be balanced with long term sequelae of therapy. Furthermore, some patients require repeated surgeries to achieve tumor control and patients with craniopharyngioma sustain severe morbidity both as a result of the tumor and the treatments. There are no recognised targeted or cytotoxic treatments available.

In this case, proven to have a somatic BRAF V600E mutation, the tumor responded promptly and progressively to monotherapy with vemurafenib. It is noteworthy that regrowth was rapid with interruption of treatment which should be considered in future cases. The molecular targeted use of the BRAF inhibitor vemurafenib and the excellent tumor response support a pathogenic role for the BRAFV600E mutation in papillary craniopharyngioma. Craniopharyngiomas have not previously been considered as treatable with cytotoxic or targeted agents. BRAF inhibitors may be an effective treatment option as part of the multimodal therapeutic armamentarium for this condition. The successful use of vemurafenib in this patient is a proof-of-concept that justifies further clinical studies and represents a potential paradigm shift in the medical management of patients harbouring a craniopharyngioma.
